# Optimizing Winter Wheat Resilience to Climate Change in Rain Fed Crop Systems of Turkey and Iran

**DOI:** 10.3389/fpls.2018.00563

**Published:** 2018-05-01

**Authors:** Marta S. Lopes, Conxita Royo, Fanny Alvaro, Miguel Sanchez-Garcia, Emel Ozer, Fatih Ozdemir, Mehmet Karaman, Mozaffar Roustaii, Mohammad R. Jalal-Kamali, Diego Pequeno

**Affiliations:** ^1^The International Maize and Wheat Improvement Center (CIMMYT), Ankara, Turkey; ^2^Sustainable Field Crops Program, Institute for Food and Agricultural Research and Technology (IRTA), Lleida, Spain; ^3^International Centre for Agricultural Research in Dry Areas, Rabat, Morocco; ^4^Bahri Dagdas International Agricultural Research Institute, Konya, Turkey; ^5^GAP Uluslararası Tarımsal Araştırma Ve Eğitim Merkezi Müdürlüğü (GAPUTAEM), Diyarbakir, Turkey; ^6^Dryland Agricultural Research Institute (DARI), AREEO, Maragheh, Iran; ^7^Global Wheat Program, The International Maize and Wheat Improvement Center (CIMMYT), Seed and Plant Improvement Institute Campus, Karaj, Iran; ^8^Socioeconomics Program, The International Maize and Wheat Improvement Center (CIMMYT), Texcoco, Mexico

**Keywords:** heading, low rainfall, reproductive stage, vegetative stage, within species diversity

## Abstract

Erratic weather patterns associated with increased temperatures and decreasing rainfall pose unique challenges for wheat breeders playing a key part in the fight to ensure global food security. Within rain fed winter wheat areas of Turkey and Iran, unusual weather patterns may prevent attaining maximum potential increases in winter wheat genetic gains. This is primarily related to the fact that the yield ranking of tested genotypes may change from one year to the next. Changing weather patterns may interfere with the decisions breeders make about the ideotype(s) they should aim for during selection. To inform breeding decisions, this study aimed to optimize major traits by modeling different combinations of environments (locations and years) and by defining a probabilistic range of trait variations [phenology and plant height (PH)] that maximized grain yields (GYs; one wheat line with optimal heading and height is suggested for use as a testing line to aid selection calibration decisions). Research revealed that optimal phenology was highly related to the temperature and to rainfall at which winter wheat genotypes were exposed around heading time (20 days before and after heading). Specifically, later winter wheat genotypes were exposed to higher temperatures both before and after heading, increased rainfall at the vegetative stage, and reduced rainfall during grain filling compared to early genotypes. These variations in exposure to weather conditions resulted in shorter grain filling duration and lower GYs in long-duration genotypes. This research tested if diversity within species may increase resilience to erratic weather patterns. For the study, calculated production of a selection of five high yielding genotypes (if grown in five plots) was tested against monoculture (if only a single genotype grown in the same area) and revealed that a set of diverse genotypes with different phenologies and PHs was not beneficial. New strategies of progeny selection are discussed: narrow range of variation for phenology in families may facilitate the discovery and selection of new drought-resistant and avoidant wheat lines targeting specific locations.

## Introduction

Water shortage (drought) and extremes in temperature (cold and heat) are common abiotic stresses worldwide (e.g., Zampieri et al., 2017). Although the wheat breeding and research community focuses on developing high yielding varieties with improved adaptation to drought and heat, annual genetic gains during the last three decades have been poor in low yielding environments (around 0.5% year, see, e.g., Lopes et al., 2012 and more recently quantified in heat stressed environments in Crespo-Herrera et al., 2017). The small amount of progress achieved to improve heat and drought adaptation and resistance is at least partially related to methodological obstacles such as: (i) the confounding effects caused by phenology (or crop stage at which the crop experiences the stress) when evaluating a diverse set of wheat genotypes and (ii) the erratic nature of weather (rainfall and temperature vary importantly from year to year). One good example of the first obstacle is that cereal genotypes of short duration will yield more than long-duration genotypes when grown under terminal drought conditions (also known as drought escape mechanism; Fischer and Wood, 1979; Mitchell et al., 1996; Lopes et al., 2014). Meiosis, the most stress sensitive stage of reproduction in wheat (Saini and Westgate, 1999) generally coincides with the booting stage (Zadoks et al., 1974) and escape of exposure to drought and heat at this stage is crucial for survival and grain set. Additionally, early flowering is an evolutionary mechanism for adaptation to warm and drought environments (Royo et al., 2014). Blum (2009) citing Mitchell et al. (1996) explained that short-term growth duration dictates moderate water-use and the escape of terminal drought stress, whereas long-duration genotypes generally have greater water use and larger deep root systems that allow deep soil moisture extraction – if indeed deep soil moisture is available. Therefore, exposure of genotypes with long and short duration to drought or heat conditions at the same developmental stage would likely reveal different effects on grain yield (GY). While the literature indicates several explanations for the better performance of short duration genotypes, exposure of these genotypes to weather variables in comparison to late duration genotypes has not been determined.

Fischer and Wood (1979) and Mitchell et al. (1996) have suggested reducing the confounding effect of phenology to explore other mechanisms of drought avoidance and tolerance. Despite these early efforts, looking at the literature, a very large number of published research studies (e.g., Araki et al., 1999; Kirigwi et al., 2007; Kuchel et al., 2007; Honsdorf et al., 2014) related to responses to drought or heat (both phenotyping and genetics) completely ignored the effect of phenology that Fischer took into consideration in his early work to improve screening methodologies for drought adaptation (Fischer and Maurer, 1978). This oversight is unfortunate and at least partially hampers our progress to increase GYs under low rainfall and warm environments. Fortunately, there have been a few significant efforts to remove the confounding effects of phenology in various wheat populations for gene discovery at the International Maize and Wheat Improvement Center (CIMMYT; Olivares-Villegas et al., 2007; Pinto et al., 2010; Lopes et al., 2013, 2015). The removal of confounding effects of phenology must be fully encouraged when developing new populations for gene discovery or other purposes related to studies of drought or heat adaptation (the evident exception would be for populations developed for the discovery of new phenology genes or related studies). In a scenario of increased temperatures in dry areas, as projected in the last IPCC report (available at: http://www.ipcc.ch/ipccreports/tar/wg2/index.php?idp=378), these considerations become even more relevant due to the increased difficulty of breeding for a combination of stresses.

Some researchers have proposed that synchronizing growth duration with the expected or predicted seasonal moisture supply takes place as the first and foremost step in breeding for water-limited environments (Blum, 2009). Although this categorization is simple, the types of rainfall patterns found in many parts of the world are becoming more erratic which makes it difficult to define the synchronization that best suits a particular environment and a specific year. Therefore, this study aims to find the most probable optimal synchronization for low rainfall winter wheat areas across years and locations. By extension, this research also aims to determine optimal plant height (PH) for this region, since height together with heading are probably the highest contributors to improved GY when not optimized and fixed by breeders (e.g., Lopes et al., 2014). For PH, both positive and negative correlations with GY have been reported depending on environmental factors. In high yielding environments (either with high rainfall or irrigated), tall genotypes will lodge and will have a GY penalty. However, in low yielding environments where PH may decrease as a response to water stress and high temperatures, increased PH (up to a certain limit) is important for creating a source of biomass and assimilates to feed grains and increase GYs (Lopes et al., 2014). Moreover, PH is not just a positive trait in terms of securing pre-anthesis reserves to filling grain but may also confer a proper pre-anthesis canopy architecture optimizing radiation interception (Miralles and Slafer, 1997).

One possible way to ensure increased plasticity to erratic weather conditions would be by planting several genotypes with various flowering times and PHs. Hypothetically, this variation would have the potential to increase resilience of the crop across years and to increase GYs. Crop variety mixtures, in which several genotypes are sown together in the same field, have been proposed as a mechanism for increasing yield stability in situations of environmental biotic and abiotic stress (Wolfe, 2000; Zhu et al., 2000; Creissen et al., 2016). This approach has proven efficiency against crop diseases (Zhu et al., 2000) and it is expected that compensation processes will also increase crop performance under low rainfall environments; however, this needs assessment.

In this context, particularly for the winter wheat rain fed areas of Turkey and Iran, a series of trials were conducted across several locations with the following objectives: (i) evaluate the contribution of cycle length to heading and PH to improve GY in rain fed winter wheat grown in low rainfall regions of both countries; (ii) explain the drought escape mechanisms in the region using environmental variables measured at key developmental stages; (iii) bridge environmental variables at key developmental stages with height and GY; (iv) define optimal phenology and PH and develop new strategies to facilitate the discovery of germplasm with adaptation and resistance to water shortage; and (v) test total production of a diverse and a mono-varietal crop setup.

## Materials and Methods

### Plant Material

One wheat population containing 250 modern wheat varieties and breeding lines (named here genotypes) from the CIMMYT International Winter Wheat Improvement Program (IWWIP) was assembled from international nurseries distributed every year (released from the early 1990s to 2011).

#### Experimental Design and Environments

Field experiments were conducted at three locations (Konya, Turkey; Diyarbakir, Turkey; and Maragheh, Iran) during four growth cycles (2012–2013, 2013–2014, 2014–2015, and 2015–2016) using an alpha lattice design with two replications. In Diyarbakir, trials were conducted at the International Agricultural Research and Training Center (GAPUTAEM), with Xerosols (Calcic Xerosols) and sown on November 20,^,^ 2012 and January 24,^,^ 2015. In Konya, trials were sown at the Bahri Dagdas International Agricultural Research Institute with Xerosols (Calcic Xerosols) on October 31,^,^ 2012, October 24,^,^ 2013, and October 20,^,^ 2014. In Maragheh, trials were established at the Dryland Agricultural Research Institute (DARI) with Xerosols (Haplic Xerosols) on October 12,^,^ 2012, October 10,^,^ 2013, and October 12, 2015 for growth cycles in 2012–2013, 2013–2014, and 2015–2016, respectively. Plot size was 1.5 m long with three rows and approximately 200 seeds per m^-2^ in 2012–2013 and 4 m long with six rows at the same seed density, for the remaining years in all locations.

#### Phenotypic Trait Evaluation

Grain yield was determined by manually harvesting the middle row of each plot in 2012–2013 and by mechanically harvesting the entire plot in 2013–2014, 2014–2015, and 2015–2016. For the manual harvest, each middle row containing straw and grain was weighed and threshed. Days to heading (DH) were determined as the number of days from the sowing date until the point when more than 50% of plants were displaying heads (Zadoks Stage 59, Zadoks et al., 1974). Days to physiological maturity (DM) were measured as the time when 50% of the spikes in a plot showed a total loss of green color, corresponding to Zadoks stage 89 (Zadoks et al., 1974). PH was determined by measuring the distance from the base of the stem to the top of the spike, excluding awns.

#### Statistical Analysis and Calculations

Analysis of variance was conducted for each year and combined years using an alpha lattice design, where genotype was considered as a fixed effect and the effect of block nested within replicate was considered as a random effect, with the PROC GLM of SAS statistical package (Sas Institute Inc., 2009). Broad-sense heritability (*H*^2^) was estimated for each trait individually to determine their reproducibility across years and within each field trial (by removing the year variance component) as: *H*^2^= *Vg*/[*Vg* + (*V*ge/*e*) +(*V*/*re*)], where *r* = number of replications, *e* = number of locations/years, *V* = error variance, *Vg* = genotypic variance, and *V*ge = genotype by locations/years interaction variance. To better understand the dependence and tradeoffs among agronomic traits and their mechanisms of operation in different wheat populations, Pearson correlation coefficients on the estimated means and associated probabilities were determined with the PROC CORR from SAS (Sas Institute Inc., 2009).

To determine the range of DH and PH where GY is maximized, all genotypes were grouped into seven categories based on DH grand means across locations and years: for DH very early (1, with average 192 days), early (2, with average 194 days), early medium (3, with average 195 days), medium (4, with average 196 days), medium late (5, with average 197 days), and late (6, with average 198 days), very late (7, with average 200 days); and for PH very short (1, with average 66 cm), short (2, with average 70 cm), short medium (3, with average 73 cm), medium (4, with average 75 cm), medium tall (5, with average 77 cm), tall (6, with average 82 cm), and very tall (7, with average 89 cm). Twenty random sets of 10 genotypes (for each set, different combinations of genotypes were randomly selected out from the 250 genotypes) were assembled for each DH and PH category. Average GY in each one of the seven phenology and height categories (average of 10 genotypes in each category) was determined for all the sets and non-linear regression equations relating GY to DH and PH were fitted using Microsoft Excel (2013). The DH and PH at which GY was maximum (optima DH and PH) were calculated as *x* = -*b*/2*a* from the quadratic functions *y* = *ax*^2^+*bx*+*c*.

To determine the effects of temperature at one of the most sensitive stages to abiotic stresses (around heading), the average mean (TA), maximum (TM), and minimum daily temperature (Tm) were calculated 20 days before (TA20BH, TM20BH, and Tm20BH) and after heading (TA20AH, TM20AH, and Tm20AH) for each genotype. Given the stress sensitivity of booting stage, the effect of temperature and rainfall at this developmental stage has a considerable impact on final GY. Moreover, booting stage and meiosis occur at different points in time for wheat genotypes differing in phenology, and the exact moment of that stage is difficult to assess in the field, particularly when several hundred wheat genotypes need to be evaluated. To predict approximate temperature during this sensitive period, average temperatures during the 20-day period before and after heading were calculated for each genotype in each location and year. To calculate the amount of rainfall before and after heading instead of the 20-day period, the entire vegetative (between sowing and heading, *R*_V EG_) and grain filling (between heading and maturity, *R*_GF_) stages were used (as shown in Araus et al., 2002). This calculation was related to the erratic patterns of rain in all locations and years where rain following just before or after the 20-day period is still available to the crop, but would not be included if calculated based on this short period. The total amount of rainfall (from sowing to maturity, *Rt*) was also calculated.

Finally, the hypothesis that growing several wheat genotypes may contribute to increase resilience of GY in environments with erratic weather patterns was tested. To do so, calculation of grain production of five wheat genotypes was compared to production of one single genotype or “mega-variety.” For this exercise, three sets of data were used: 250 modern winter wheat varieties and breeding lines described above and data generated from international nurseries distributed worldwide by CIMMYT, the 23rd ESWYT (Elite Selection Wheat Yield Trials), and the 21st SAWYT (Semi-Arid Wheat Yield Trial). The data can be found at: https://www.cimmyt.org/international-wheat-improvement-network-iwin/. First, environments were named “training environments” if used for selection of genotypes based on mean GY and “testing environments” if used to test performance of genotypes previously selected in the “training environments.” Then, one or five genotypes with the highest mean yield over six “training environments” were tested for their performance in two other random environments or “testing environments.” In total, 28 combinations of environments were developed using six random “training environments” and two “testing environments.” For each combination, total production of different selections was calculated: (i) total grain production of one genotype grown in two “testing environments” (total of 20 ha), and previously showing the highest mean yield, in six “training environments” (or “mega-variety”); (ii) total grain production of five genotypes grown in two “testing environments” (total of 20 ha) previously showing the top five highest mean yields in six “training environments”; and (iii) total grain production of the genotype showing the highest mean yield in all eight environments in a total of 20 ha.

The total grain production resulting from growing one genotype in *e* “testing environments” (*TiGP*) was calculated by multiplying GY in the *i*th environment (*iGY*) in gm^-2^ to total production in tonnes in 10 ha by multiplying by 0.1 (100,000 m^2^ divided by 1,000,000 g) and summing grain production of that particular genotype in *e* “testing environments,” as indicated in the equation below.

$$\begin{array}{c} TiGP\;=\sum_{i=1}^{e2}\left(0.1*iGY\right) \end{array}$$

The total grain production resulting from growing five genotypes in “testing environments” (*TiiGP*) was calculated as the sum grain production of each of the five genotypes in 2 ha (corresponding to five genotypes in 10 ha). The grain production of the *i*th genotype was calculated by multiplying GY of the *i*th genotype (*iGY*) in gm^-2^ to total production in tonnes in 2 ha by multiplying by 0.02 (20,000 m^2^ and divided by 1,000,000 g) and summing grain production for the five genotypes, as indicated in the equation below.

$$\begin{array}{c} TiiGP\;=\sum_{i=1}^{5}\left(0.02*iGY\right) \end{array}$$

Finally, *TiiGP* resulting from each of the *e* “testing environments” were summed together to obtain total production of five genotypes in 10 ha and in *e* environments. Analysis of variance was conducted with all 28 combinations (treating combinations as replicates) to determine if differences between production of one genotype or five genotypes were significant.

The same exercise was carried out in two other sets of data from the 23rd ESWYT (grown in 64 environments, 32 were used as “training environments” and other 32 used as “testing environments”) and 21st SAWYT (grown in 66 environments, 33 were used as “training environments” and other 33 used as “testing environments”). For these two sets of data, a sample of 10 random combinations of “training” and “testing environments” was used and the number of environments in equation *TiGP* was increased to 32 (total of 320 ha) and 33 (total of 330 ha) for the 23rd ESWYT and 21st SAWYT, respectively.

## Results

### Characterization of Environmental Conditions of Field Trials

Meteorological data of all trials conducted in Turkey and Iran are shown in **Table 1**. Environments where trials were conducted have shown typical conditions of winter wheat low-moderate rainfall environments (mega-environment 12, Braun et al., 2010). Experiments in Diyarbakir showed the highest minimum, maximum, and average temperatures and total rainfall (**Table 1**). These environments were also the most productive. Similar temperatures were observed in Konya and Maragheh throughout all seasons, although Konya tended to be warmer with slightly higher average and maximum temperatures (**Table 1**). Overall, Tm and Rainfall significantly explain variations in GY across locations and years (*R*^2^ of 65 and 62%, respectively, when regressing Tm and Rainfall with GY in all eight environments using data in **Table 1**).

**Table 1:** Meteorological data of the field trials conducted in Konya and Diyarbakir (Turkey) and Maragheh (Iran) between 2012 and 2016.

LOC	Harvest Year	ENV#	Tm °C	TA °C	TM °C	TMO	Rain mm	GY (g m^-2^)	DH (days)	CORR GY-DH	CORR GY-PH
Diyarbakir	2013	1	8.1	12.5	17.2	41.8	578	384	154	**–0.37^∗∗∗^**	–0.09, NS
Konya		2	4.3	10.8	17.0	34.9	254	339	191	**–0.34^∗∗∗^**	**0.15^∗∗^**
Maragheh		3	3.5	8.2	12.8	34	282	180	228	0.03, NS	**0.14^∗^**
Konya	2014	4	2.3	9.5	16.3	37	225	136	205	0.05, NS	**0.50^∗∗∗^**
Maragheh		5	2.3	7.2	12.2	35	209	223	234	**–0.37**^∗∗∗^	**0.27^∗∗∗^**
Diyarbakir	2015	6	7.3	13.8	20.3	43.4	556	415	113	**–0.47**^∗∗∗^	0.10, NS
Konya		7	2.5	8.4	14.7	33.3	288	295	214	0.01, NS	**0.29^∗∗∗^**
Maragheh	2016	8	3.5	8.3	13.2	35.5	338	287	227	**–0.22^∗∗∗^**	–0.03, NS

*Average minimum temperature (Tm), average mean temperature (TA), average maximum temperature (TM), maximum temperature observed (TMO), rainfall (Rain) from sowing to physiological maturity, average grain yield (GY), and cycle length to heading (DH) are presented. Pearson correlation coefficients between GY and DH (CORR GY-DH) and between GY and PH (CORR GY-PH) are shown for each trial. ^∗∗∗^, significant at *p* < 0.0005; ^∗∗^, significant at *p* < 0.005; ^∗^, significant at *p* < 0.05; NS, non-significant. Significant correlations are shown in bold values.*

In more detail, **Supplementary Figures S1A–H** show the window of variation for heading date and maturity in each location and year tested for this study together with daily rainfall and temperature. Overall, these environments are characterized by cold winters, progressively higher temperatures, and a decrease in rainfall at around heading time continuing to maturity (**Supplementary Figures S1A–H**). In all trials (locations and years), heading of all genotypes occurred when temperatures were rising (**Supplementary Figures S1A–H**). On a daily basis, rainfall patterns were erratic across locations and years (**Supplementary Figures S1A–H**). However, overall, the amount of rainfall at the grain filling stage (*R*_GF_) was on average (across locations and years) around 12% of the amount falling at the vegetative stage (**Table 2**).

**Table 2:** Basic statistics [heritability (*H*^2^), standard error of mean (SEM), standard error of difference (SED), least significant difference (LSD), average (avg), maximum (max), and minimum (min)] of agronomic and weather traits measured in a winter wheat population (250 varieties and breeding lines) grown in eight trials between 2012 and 2016 in Turkey and Iran.

	DH	DM	GFD	GY	PH	TA_20BH_	TA_20AH_	TM_20BH_	TM_20AH_	Tm_20BH_	Tm_20AH_	*R*_V EG_	*R*_GF_	*R_t_*
*H*^2^	0.91	0.83	0.47	0.48	0.89	0.80	0.76	0.79	0.65	0.73	0.82	0.70	0.67	0.00
SEM	0.52	0.83	0.78	625	5.5	0.03	0.03	0.05	0.05	0.03	0.02	1.7	2.5	1.2
SED	1.0	1.3	1.2	35	3.3	0.2	0.2	0.3	0.3	0.2	0.2	7.8	2.2	1.5
LSD	2.0	2.6	2.5	71	6.7	0.5	0.5	0.6	0.6	0.5	0.4	3.6	4.5	3.1
avg	196	231	35	261	76	15	18	22	26	7.8	10	251	39	291
max	205	239	38	335	100	16	20	24	27	9.3	12	262	45	293
min	191	226	32	144	58	14	17	20	25	7.1	9.6	246	31	288
G	^∗∗∗^	^∗∗∗^	^∗∗∗^	^∗∗∗^	^∗∗∗^	^∗∗∗^	^∗∗∗^	^∗∗∗^	^∗∗∗^	^∗∗∗^	^∗∗∗^	^∗∗∗^	^∗∗∗^	NS
E	^∗∗∗^	^∗∗∗^	^∗∗∗^	^∗∗∗^	^∗∗∗^	^∗∗∗^	^∗∗∗^	^∗∗∗^	^∗∗∗^	^∗∗∗^	^∗∗∗^	^∗∗∗^	^∗∗∗^	^∗∗∗^
GxE	^∗∗∗^	^∗∗∗^	^∗∗∗^	^∗∗∗^	^∗∗∗^	^∗∗∗^	^∗∗∗^	^∗∗∗^	^∗∗∗^	^∗∗∗^	^∗∗∗^	^∗∗∗^	^∗∗∗^	^∗∗∗^
E Avg														
1	155	195	40	384	92	14	17	21	26	7.1	9.5	384	105	489
2	191	225	34	339	78	15	17	22	24	5.9	9.3	217	34	251
3	228	268	40	120	76	12	17	16	24	7.6	10	272	6.7	279
4	205	243	37	136	57	16	19	24	26	7.8	9.8	150	38	188
5	234	268	34	221	66	15	18	20	25	9.5	11	206	3.0	209
6	113	145	31	311	99	18	21	26	29	9.9	12	209	42	252
7	214	244	30	295	67	14	17	23	24	6.2	9.1	217	23	240
8	227	263	36	287	72	14	19	20	26	8.4	1.0	355	61	417

*Analysis of variance with trial (E), genotype (G), and genotype by trial interaction (G × E) effects are shown. Average per environment is shown (E AVG) for each trait. Environment (E) 1–8 correspond to locations and years as shown in **Table 1**; days to heading (DH), days to maturity (DM), grain filling duration (GFD), grain yield (GY), and plant height (PH) measured in a population containing 250 varieties and breeding lines grown in eight trials between 2012 and 2016 in Turkey and Iran. Exposure of genotypes to weather conditions is shown: average temperature 20 days before (TA_*20BH*_) and after heading (TA_*20AH*_); average maximum temperature 20 days before (TM_*20BH*_) and after heading (TM_*20AH*_); average minimum temperature 20 days before (Tm_*20BH*_) and after heading (Tm_*20AH*_); rain from sowing to heading (*R*_*VEG*_); rain from heading to maturity (*R*_*GF*_); and total rain from sowing to maturity (*R_*t*_*). ^∗∗∗^, significant at *p* < 0.0005; ^∗∗^, significant at *p* < 0.005; ^∗^, significant at *p* < 0.05; NS, non-significant.*

### Contributions of DH and PH to Grain Yield in Turkey and Iran

Since one of the main objectives of this study is to determine optimal DH and PH to maximize GY, correlations between these two traits were calculated per trial (**Table 1**). Significant negative correlations between DH and GY were reported in Diyarbakir 2013, Konya 2013, Maragheh 2014, Diyarbakir 2015, and Maragheh 2016 (**Table 1**). Correlations (linear associations) between GY and DH in other trials were not significant (**Table 1**). PH correlated positively with GY (and statistically significant) in Konya 2013, Maragheh 2013, Konya 2014, Maragheh 2014, and Konya 2015, being those also the environments with lower rainfall (**Table 1**). Correlations between GY and PH in other trials were not significant (**Table 1**). Using the means of each genotype across locations and years, DH and PH correlated negatively with GY (*r* = -0.40 and *r* = -0.15; **Table 3**).

**Table 3:** Correlation matrix with Pearson correlation coefficient and associated probability of overall weather and agronomic traits as means across all tested trials in Turkey and Iran.

*N* = 250	DM	GFD	GY	PH	TA_20BH_	TA_20AH_	TM_20BH_	TM_20AH_	Tm_20BH_	Tm_20AH_	*R*_V EG_	*R*_GF_
DH	**0.87**	**–0.49**	**–0.40**	**–**0.04	**0.97**	**0.95**	**0.96**	**0.93**	**0.96**	**0.96**	**0.93**	**–0.93**
	**^∗∗∗^**	**^∗∗∗^**	**^∗∗∗^**	NS	**^∗∗∗^**	**^∗∗∗^**	**^∗∗∗^**	**^∗∗∗^**	**^∗∗∗^**	**^∗∗∗^**	**^∗∗∗^**	**^∗∗∗^**
DM		**–**0.01	**–0.29**	**–**0.03	**0.85**	**0.84**	**0.85**	**0.82**	**0.83**	**0.86**	**0.79**	**–0.78**
		NS	**^∗∗∗^**	NS	**^∗∗∗^**	**^∗∗∗^**	**^∗∗∗^**	**^∗∗∗^**	**^∗∗∗^**	**^∗∗∗^**	**^∗∗∗^**	**^∗∗∗^**
GFD			**0.31**	0.02	**–0.46**	**–0.46**	**–0.45**	**–0.45**	**–0.49**	**–0.43**	**–0.49**	**0.50**
			**^∗∗∗^**	NS	**^∗∗∗^**	**^∗∗∗^**	**^∗∗∗^**	**^∗∗∗^**	**^∗∗∗^**	**^∗∗∗^**	**^∗∗∗^**	**^∗∗∗^**
GY				**–0.15**	**–0.39**	**–0.42**	**–0.39**	**–0.40**	**–0.40**	**–0.36**	**–0.37**	**0.36**
				**^∗∗^**	**^∗∗∗^**	**^∗∗∗^**	**^∗∗∗^**	**^∗∗∗^**	**^∗∗∗^**	**^∗∗∗^**	**^∗∗∗^**	**^∗∗∗^**
PH					**–**0.04	**–**0.02	**–**0.03	**–**0.03	**–**0.06	**–**0.06	**–**0.10	0.08
					NS	NS	NS	NS	NS	NS	NS	NS
TA_20BH_						**0.95**	**0.94**	**0.97**	**0.98**	**0.94**	**–**0.11	**–0.39**
						**^∗∗∗^**	**^∗∗∗^**	**^∗∗∗^**	**^∗∗∗^**	**^∗∗∗^**	NS	**^∗∗∗^**
TA_20AH_							**0.93**	**0.93**	**0.96**	**0.93**	**–**0.10	**–0.38**
							**^∗∗∗^**	**^∗∗∗^**	**^∗∗∗^**	**^∗∗∗^**	NS	**^∗∗∗^**
TM_20BH_								**0.93**	**0.96**	**0.93**	**–**0.10	**–0.38**
								**^∗∗∗^**	**^∗∗∗^**	**^∗∗∗^**	NS	**^∗∗∗^**
TM_20AH_									**0.95**	**0.95**	**–**0.09	**–0.40**
									**^∗∗∗^**	**^∗∗∗^**	NS	**^∗∗∗^**
Tm_20BH_										**0.93**	**–0.14**	**–0.40**
										**^∗∗∗^**	**^∗∗^**	**^∗∗∗^**
Tm_20AH_											**–**0.12	**–0.36**
											NS	**^∗∗∗^**
*R*_V EG_												**–0.94**
												**^∗∗∗^**
												

*All traits were measured in a wheat population containing 250 varieties and breeding lines grown in eight trials between 2012 and 2016 in Turkey and Iran. Days to heading (DH), days to maturity (DM), grain filling duration (GFD), grain yield (GY), and plant height (PH). Exposure of genotypes to weather conditions is shown: average temperature 20 days before (TA_*20BH*_) and after heading (TA_*20AH*_); maximum temperature 20 days before (TM_*20BH*_) and after heading (TM_*20AH*_); minimum temperature 20 days before (Tm_20*BH*_) and after heading (Tm_*20AH*_); rainfall between sowing and heading (*R*_*VEG*_); and rainfall between heading and maturity (*R*_*GF*_). ^∗∗∗^, significant at *p* < 0.0005; ^∗∗^, significant at *p* < 0.005; ^∗^, significant at *p* < 0.05; NS, non-significant. Significant correlations are shown in bold values.*

### Characterization of Temperature 20 Days Before and After Heading and Rainfall at the Vegetative and Grain Filling Stages

Average, maximum, and minimum temperatures during the 20-day period before and after heading (TA20BH, TA20AH, TM20BH, TM20AH, Tm20BH, and Tm20AH, respectively) were calculated in each trial and across all trials (**Table 2**). Different genotypes (varying in DH for more than 10 days, see maximum and minimum across locations and years in **Table 2**) were consistently exposed to different temperatures in the time period of 20 days before and after heading (**Table 2**) as shown by significant “genotype” effects and medium to high heritability (0.65–0.82) across trials for TA20BH, TA20AH, TM20BH, TM20AH, Tm20BH, and Tm20AH. Moreover, for total rainfall at the vegetative and grain filling stages (*R*_V EG_ and *R*_GF_), significant “genotype” effects were observed across locations and years and heritability of 0.67–0.70 showing that different genotypes are exposed to different amount of rainfall in a consistent manner across locations and years. Moreover, the “genotype” effect for total rainfall (*R_t_*, corresponding to the sum of *R*_V EG_ + *R*_GF_) across locations and years was not significant with 0.00 reproducibility (*H*^2^) showing that the total amount of rainfall received by all genotypes was the same.

**FIGURE 1:**
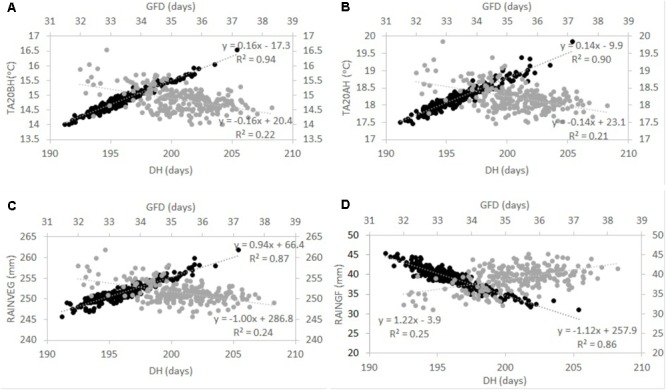
Tradeoff between days to heading (DH) and grain filling duration (GFD) as explained by average temperature 20 days before (TA20BH) and after (TA20AH) heading (**A** and **B**, respectively), and rainfall at the vegetative (RAIN_V EG_) and grain filling (RAINGF) stages (**C** and **D**, respectively) in a winter wheat population grown in Turkey and Iran between 2012 and 2016. Grand means across environments for each genotype are shown. Dark circles represent DH and gray circles represent GFD (shown in secondary axes). Regression equations and *R*^2^ are shown. Correlations are all significant at *P* < 0.005 as shown in **Table 3**.

After the overall analysis revealed that genotypes with variable phenology were exposed to different temperatures and rainfall at specific stages of development, simple correlations were calculated (**Table 3**). In the type of environments and wheat genotypes tested herein, GY correlated negatively with DH (*r* = –0.40) and DM (*r* = –0.29) and positively with grain filling duration (GFD, *r* = 0.31; **Table 3**). Moreover, DH and DM correlated positively with temperature 20 days before and after heading (*r* > 0.90 and *r* > 0.80, respectively). The amount of rainfall at the vegetative stage (*R*_V EG_) correlated positively with DH and DM (*r* = 0.93 and *r* = 0.79, respectively), whereas *R*_GF_ correlated negatively with DH and DM (*r* = -0.93 and *r* = -0.78, respectively; **Table 3**). Temperature 20 days before and after heading and *R*_V EG_ correlated negatively with GY (*r* > -0.36 and *r* = -0.37); however, *R*_GF_ correlated positively with GY (*r* = 0.36; **Table 3**). Correlations between temperature and GY were not consistent when each environment was taken individually (**Supplementary Table S1**). Sometimes temperature had a positive effect (e.g., Diyarbakir 2013 and Maragheh 2013); however, in Maragheh 2014, Diyarbakir 2015, and Maragheh 2016, temperature had a negative effect on GY. Correlations per locations and years of *R*_V EG_ and *R*_GF_ with GY when significant were consistently negative and positive, respectively (**Supplementary Table S1**). To better illustrate the tradeoffs between DH and GFD as explained by *R*_GF_, *R*_V EG_, and temperature, regressions are represented in **Figure 1**.

### Calculating Optimal DH and PH to Maximize Grain Yield in a Changing Environment

**FIGURE 2:**
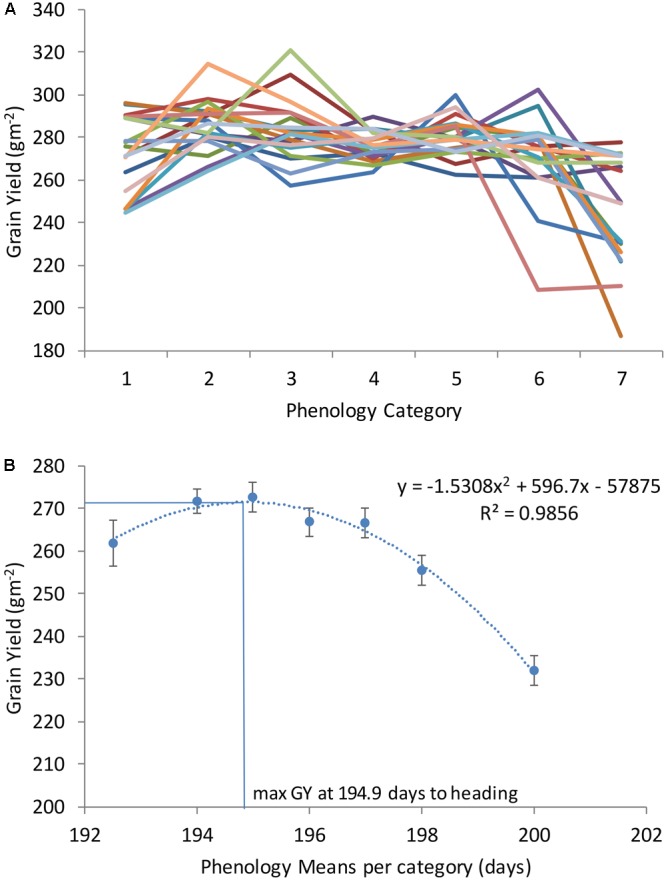
Grain yield (GY) of seven phenology categories in a winter wheat population of 250 varieties and breeding lines grown in Turkey and Iran (1, very early; 2, early; 3, early-medium; 4, medium; 5, medium late; 6, late; and 7, very late). Twenty sets of 10 genotypes were randomly selected of each phenology category to model phenology and associated grain yield **(A)**. Average grain yield associated with each phenology category was calculated as an average of all sets (assembled with 10 genotypes of each category in **B**).

**FIGURE 3:**
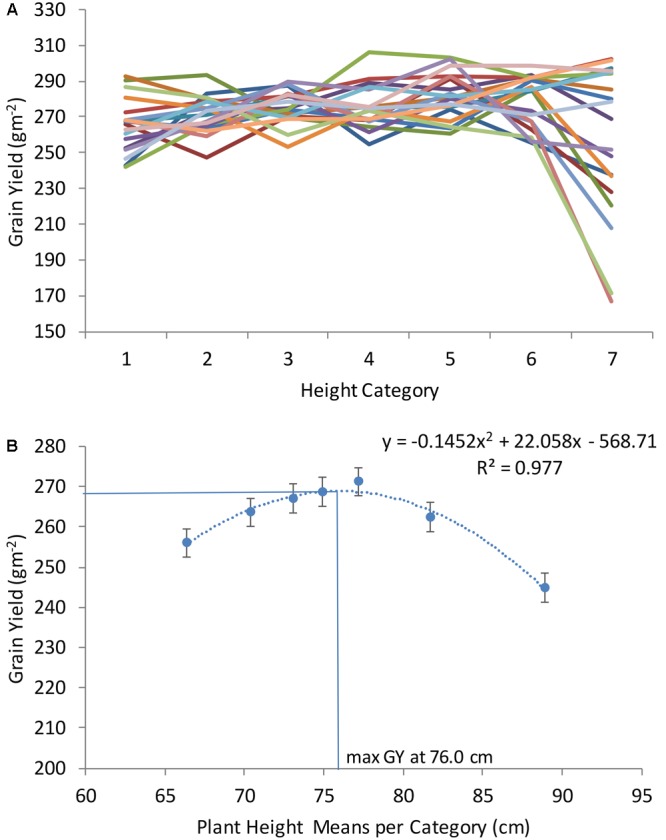
Grain yield (GY) of seven plant height categories in a winter wheat population of 250 varieties and breeding lines grown in Turkey and Iran (1, very short; 2, short; 3, short-medium; 4, medium; 5, medium tall; 6, tall; and 7, very tall). Twenty sets of 10 genotypes were randomly selected of each height category to model height and associated grain yield **(A)**. Average grain yield associated with each height category was calculated as an average of all sets (assembled with 10 genotypes of each category in **B**).

With the objective of finding the DH and PH that maximize GY across locations and years, all genotypes were classified according to seven DH and PH categories (as described in Section “Materials and Methods”). After categorization, random genotypes were selected from each category and used to plot with GY as shown in **Figures 2, 3**. Each line in **Figures 2A, 3A** corresponds to average GY of 10 genotypes randomly selected and falling within seven DH and PH categories. This exercise was repeated 20 times corresponding to 20 lines in **Figures 2A, 3A** (each time using different genotypes from the entire population consisting of 250 winter wheat breeding lines and varieties). The quadratic function shown in **Figure 2B** and representing the relationship DH with GY revealed that maximum yields were obtained at 194.9 DH. The maximum of the function in each location was also calculated for DH and is shown in **Supplementary Table S2**. For PH, the quadratic function shown in **Figure 3** revealed that maximum yields were obtained at 76.0 cm average height across all locations and years (**Figure 3B**). Moreover, the maximum of the quadratic function was also calculated for PH, in each location and is shown in **Supplementary Table S2**. Finally, one modern wheat breeding line with optima DH and PH was selected for each location which can be used in testing blocks for calibration during selection procedures (**Supplementary Table S3**). Moreover, major functional genes are shown for the selected genotypes (**Supplementary Table S3**). Since optima DH and PH were different in each location (**Supplementary Tables S2, S3**), the differences in GY obtained by selecting for specific adaptation to each location or by selecting for adaptation to all locations together were calculated (**Supplementary Table S4**). These calculations have shown, that by selecting wheat breeding lines with optima DH and PH in each location provided on average an increase of 18.6% GY compared to selection based on all locations and years (average of %CHANGE_GY in **Supplementary Table S4**).

### Testing Resilience and Total Production of a Diverse and a Mono-Varietal Crop Setup

**FIGURE 4:**
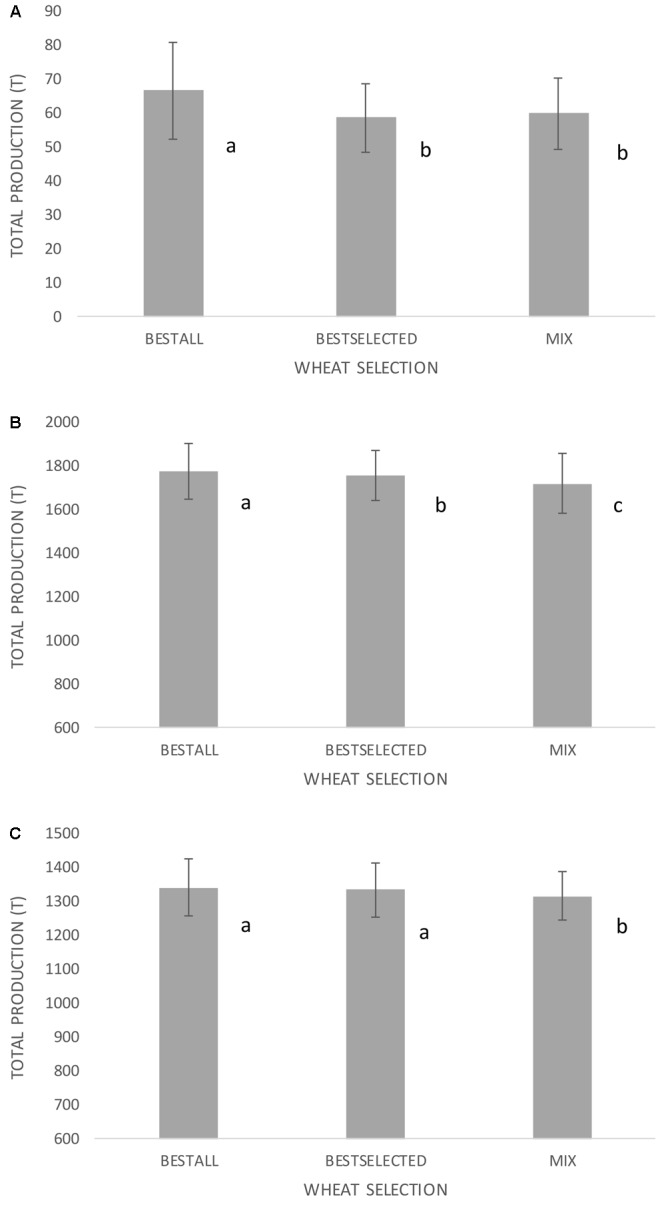
Grain production using three sets of international trials including **(A)** Turkey and Iran for 4 years (eight trials with low yielding environments) in a total of 20 ha, **(B)** 64 trials across the globe for the international nursery 23rd ESWYT (Elite Selection Wheat Yield Trials- high yielding environments) in a total of 320 ha, and **(C)** 66 trials across the globe for the international nursery 21st SAWYT (Semi-Arid Wheat Yield Trial – low yielding environments) in a total of 330 ha. BESTALL is the actual best wheat genotype in the whole set of environments (based on grand means across all environments), BESTSELECTED corresponds to the best wheat genotype selected in a subset of training environments (based on grand means across half of the environments), and MIX corresponds to the best five selected genotypes in a subset of training environments and grown together in 2 ha plots side by side. Different letters indicate significant differences using LSD test at *p* < 0.05. Error bars indicate SD across random sets of environments.

Three sets of germplasm and data were used to test if several wheat varieties grown together would buffer the effects of erratic weather patterns: (i) wheat population used herein (grown in eight environments); (ii) 23rd ESWYT (grown in 64 environments most of them irrigated and high yielding); and (iii) 21st SAWYT (grown in 66 semi-arid environments with little or no irrigation and low yielding). One of the objectives of this exercise was to identify the selection methodology that will permit the identification of the best possible genotype(s) for a specific type of environment. Therefore, genotypes for (i)–(iii) were selected based on GY performance in “training environments” and then the selected genotypes were evaluated in a set of “testing environments,” which correspond to the GY shown in **Figures 4A–C**. This scenario would simulate what generally happens when breeders make a selection of new promising wheat lines based on their best performance (highest GY) in international trials, and farmers adopt those selected lines. Second, this exercise aimed to test whether instead of using one single wheat genotype, five genotypes would boost resilience and total grain production across environments and years. The results revealed that the best genotype in monoculture always demonstrated higher production than a group of the five best genotypes provided that the number of environments was sufficient enough to find the genotype showing the highest yield (**Figure 4A**). However, the best selected genotype in “six training environments” showed similar grain production to five genotypes. Given the fact that the number of trials conducted for this population was relatively low, the same hypothesis was tested in spring wheat where data from international nurseries are available at CIMMYT. In the two sets of data for the 23rd ESWYT and 21st SAWYT, always the selection of the best unique genotypes gave the highest GY and the group of five genotypes showed no benefit comparatively. In the 23rd ESWYT (**Figure 4B**), the best-selected genotype in 32 training environments had lower GY than the best-selected unique genotype across all 64 environments. However, in the 21st SAWYT (**Figure 4C**), the best-selected unique wheat genotype in 33 training environments had similar GY to the best-selected genotype in all 66 field trials.

## Discussion

### Contributions of DH and PH to Improve Grain Yield in Rain Fed Winter Wheat Grown in Turkey and Iran

Manipulation of DH and PH in wheat has probably had the highest impact on wheat GY globally and both are also responsible for the adoption of wheat in a wide range of environments (Reynolds and Bourlag, 2006; Lopes et al., 2014, 2015; Royo et al., 2017). Cycle length to heading and PH together may explain up to 68% of GY variation in low yielding environments (Lopes et al., 2014) although this largely depends on the genetic background of the wheat genotypes, range of variation of DH and PH, and the environments being tested. Given the importance of these two traits and the simplicity of detection in the field during selection of progenies in breeding programs, the optimal expression of these traits was evaluated and calculated to maximize GY. For this study, DH and PH were determined in a series of experimental field trials in three locations for 4 years. This wheat population was intentionally designed for expressing a wide range of variation for phenology to understand more about how different mechanisms of adaptation and stress escape of early and late genotypes in rain fed winter wheat areas of Turkey and Iran. Cycle length to heading and GY correlated negatively and significantly in most trials tested. However, PH correlated positively and significantly with GY in most field trials, but overall PH correlated negatively with GY when means across locations and years for each genotype were used (but with weak Pearson Correlation coefficient *r* = -0.15). These results are in accordance with previous observations because weather conditions partially contribute to changes in optimal DH and PH, and PH contributions to GY (Lopes et al., 2012, 2013). For the type of locations and years studied (winter wheat region of Turkey and Iran), some level of earliness has been identified as important to escape periods of water shortage during grain filling. To better explain the mechanisms related to this response, the environmental conditions to which different DH and PH winter wheat genotypes are exposed are analyzed below.

### Drought Escape Mechanisms in Winter Wheat Grown Under Low Rainfall Regions of Turkey and Iran

Grain yield across trials was primarily and significantly affected by rainfall and Tm as shown by the significant correlations between GY and the former weather variables. As described in Section “Introduction,” Fischer and Wood (1979) and Mitchell et al. (1996) highlighted the discrimination against long-duration genotypes in poor-yielding environments, and the resulting confounding effects hindering the discovery of genotypes with drought avoidance and resistance. While these confounding effects have been reported and cited in the literature, the mechanisms associated with this response are poorly understood in the context of winter wheat rain fed regions of Turkey and Iran. When comparing different wheat genotypes (particularly with variable phenology) in one specific field trial, it is assumed that all genotypes are exposed to the same environmental conditions. While this is correct if an average of all environmental traits is taken from sowing to harvest, it may not be when temperature and rainfall are determined at specific developmental stages. Since different genotypes attain a certain developmental stage at different points in time, weather data were collected and calculated for each genotype at key developmental stages. Specifically, temperature and rainfall were quantified and evaluated for the period 20 days before and after heading in a series of genotypes. The results presented here show that genotypes differing in their phenology were exposed to different temperatures and rainfall 20 days before and after heading. Primarily, DH positively correlated with temperature TA, TM, and Tm 20 days before and after heading. On average across locations and years, the effect of delaying heading by 1 day resulted in an increase of 0.16 and 0.14°C in temperature 20 days before and after heading, respectively (as shown by the regression line in **Figures 1A,B**). This means that two genotypes with a 10-day difference in heading may be exposed to a difference in temperature of up to 1.6 and 1.4°C (on average across locations and years) 20 days before or after heading. This increase in temperature of late genotypes led to shorter grain filling duration (**Figures 1A,B**) as shown by a negative correlation of temperature with GFD and DH with GFD. This is in agreement with Bruckner and Frohberg (1987) where increasing temperature during grain filling in different genotypes tended to stop grain growth prematurely and to hasten physiological maturity.

In the field trials conducted here, even if the total amount of rainfall from sowing to maturity received by each genotype was similar, the distribution of this rainfall changed across genotypes at a specific growth stage. Non-significant “genotype” effects showed this for *Rt* (total rainfall from sowing to maturity) in the analysis of variance across locations and years and significant “genotype” effects for *R*_V EG_ (total rainfall during the vegetative stage for each genotype) and *R*_GF_ (total rainfall during the grain filling stage for each genotype) across locations and years. Moreover, *R*_V EG_ and *R*_GF_ correlated positively and negatively with DH (**Table 3**), respectively (*Rt* did not show “genotype” effects and was not included for correlation analysis). On average across locations and years, the effect of delaying heading by 1 day resulted in an increase of 0.94 mm in *R*_V EG_ (as shown by the regression line in **Figure 1C**) and a decrease of 1.12 mm in *R*_GF_. This shows that on average across the type of locations and years tested, late genotypes were exposed to more rainfall during the vegetative stage, but were also exposed to less rainfall at the grain filling stage compared to earlier genotypes, and as demonstrated by the regression lines of both periods (**Figures 1C,D**). In conclusion, increased temperature at around heading and less rainfall during the grain filling period explain the lower success of late winter wheat genotypes in the type of locations and years tested.

### Bridging Temperature and Rainfall Exposures of Different Genotypes With Height and GY

Temperature at around heading and *R*_V EG_ and *R*_GF_ across genotypes were not correlated with PH, although PH had an effect on GY as shown by positive correlations in several field trials. This indicated that mechanisms through which PH affected GY in these environments were not driven by the differences observed in temperature and rainfall at a specific developmental stage across genotypes. However, the correlation of PH with GY across genotypes was a function of total rainfall in each year and location, with correlations trending negative to null with high rainfall and correlation trending positive with low rainfall. This result is in accordance with Lopes et al. (2014) where the success of taller wheat genotypes was associated with low yielding low rainfall environments. Other studies have explained the success of taller wheat varieties in dry environments through longer coleoptiles and larger roots systems compared with the semi-dwarfs, where deep sowing ensures contact with available soil moisture (Rebetzke and Richards, 2000), improved pre-anthesis radiation interception (Miralles and Slafer, 1997), and decreased canopy temperature (Lopes et al., 2012a). Mathews et al. (2006) analyzed 81 trials around the world and concluded that tall and semi-dwarf lines had similar adaptation to stressed environments and showed the highest GY. It is also worth noting that tall varieties may be desirable for reasons other than high yield, including high biomass and longer straw lengths (can be used as animal feed).

### Fitting DH and PH to the Environment as a Major Goal to Increase Adaptation and Maximize Grain Yield

One challenge for future wheat breeding is to modify the reproductive stage timing to suit local climatic conditions while maintaining or even increasing yield potential (Langer et al., 2014). In most instances, wheat breeders have been guided by a set of highly productive wheat check lines or genotypes that in principle have optimal DH and PH. However, optimal synchronization has not always been achieved in rain fed winter wheat. In this study, a major objective was to define optimal DH and PH for winter wheat grown under low rainfall cold temperature (ME12, Braun et al., 2010) and fit the most sensitive stages of crop development to the most favorable time and weather conditions of the wheat cycle. A method of calculation was used to categorize genotypes into different DH and PH groups, and random genotypes were selected from each category to determine how these influenced GY. Optima DH and PH were calculated across all locations and years together and GY was maximized at 194.9 DH and at 76.0 cm PH. However, optima DH and PH varied with location. Specifically, Diyarbakir is warmer and wetter (higher temperatures and total amount of rainfall) than Konya and Maragheh and earlier genotypes are better adapted to Diyarbakir. Konya and Maragheh are cooler and winter wheat (later genotypes) is better adapted to these regions. These specificities and requirements in each location studied herein showed that to improve GY, selection must target various optima DH and PH. This was shown by a 18.6% increase in GY when selection targeted optima DH and PH obtained from Diyarbakir, Konya, and Maragheh separately compared to targeting one overall (across all locations and years) optima DH and PH. A wheat breeding line with optima DH and PH for each location was selected and these can be used as checks in the field in demonstration plots for calibration during selection procedures (**Supplementary Table S2**). Moreover, in international breeding programs targeting many different regions, multiple optimal DH and PH ranges must be identified according to the target regions to avoid the risks of limiting the amount of progress in those regions.

### Testing Resilience and Total Production of a Diverse and a Mono-Varietal Crop Setup

Crop biodiversity has been proposed as a possible solution to the vulnerability of monoculture crops to disease (Zhu et al., 2000) and climate variability (Lin, 2011). Crop diversity and the coexistence of multiple species have been used as an example for improved resilience (Lin, 2011). However, limited information is available on the resilience within species diversity to increase resilience of wheat systems to climate change. In theory, it would be beneficial for wheat production to have a set of a few highly productive wheat genotypes grown in one particular farm over a period of years to increase wheat resilience through compensation and adaptation mechanisms of different traits. This hypothesis was tested for this study by selecting the best wheat genotypes in sets of environments, and comparing the best selected genotype in monoculture (hypothetical defined in 10 ha) and in groups of five genotypes grown in 2 ha per genotype (in a total of 10 ha). The results revealed that the best-selected genotype in monoculture always demonstrated higher production than a group of the five best genotypes. The results were unexpected given that different phenology, PH, and other traits in the diverse set (five genotypes) would help minimize the losses caused by one “bad year” for one specific genotype. Probably, crop variety mixtures are not the solution for water and heat stresses in the tested environments, given the fact these stresses may occur too late in the crop cycle, when crop plasticity (in terms of tillering, for example) is already accomplished. It may have more sense for environments when water stress occurs during the first stages of crop cycle and this needs further testing. Moreover, this hypothesis was tested for this study by also using data from international nurseries released each year by CIMMYT. This data were used to select the five best genotypes in 32 and 33 training environments (used for genotype selection) for the ESWYT and SAWYT, respectively, and then by testing those selected genotypes in other 32 and 33 testing environments (not used for genotype selection). Using a diverse setup of five genotypes always resulted in lower yields compared to a monoculture with the highest yielding genotype and this was mainly due to the fact that the total crop area also contained lower yielding genotypes. Moreover, the best-selected genotype in a set of 33 training environments was at least for the 21st SAWYT the best genotype in 33 testing environments. However, for the 23rd ESWYT, the best-selected genotype in the training environments was not always the best genotype in the testing environments. This indicated a gap between highest potential GY attainable, i.e., if the highest GY genotype can be identified and adopted and genotypes actually obtained by selection and eventually adopted by farmers. To close this gap, more than 30 training environments must be used to ensure that the best-selected variety will actually perform well in a wide range of environments. For this reason, international nurseries at CIMMYT are planted in at least 60 environments to ensure the best possible selection.

## Conclusion

For many years, researchers have realized the slow yield progress of less productive wheat areas in the world where irrigation is not an option or where water resources are becoming scarce. To date, research efforts to tackle this bottleneck have not been sufficient to reverse the slow progress. The results presented from this study helped to define the aspects of wheat breeding that need attention, and are preventing our progress in environments with low rainfall and cold temperature in Turkey and Iran. To increase genetic gains in rain fed winter, wheat the adoption of phenology that synchronizes with rainfall distribution typical from a specific region at the vegetative and grain filling stages is necessary. It was shown that genotypes with different phenologies received different rainfall and temperatures at a specific key developmental stage. Late-duration genotypes received more rainfall during the vegetative stage and less rainfall at the grain filling stage compared to short-duration genotypes. Moreover, the late-duration genotypes were exposed to higher temperature at around heading, and both rainfall and temperature shortened the grain filling duration and penalized yields. Optimal DH and PH that maximize GY in rain fed winter wheat areas of Turkey and Iran have now been defined. After that, parental lines with similar synchronization with rainfall and temperature (similar optimal heading dates) but differing for other traits should be crossed. Alternatively, even if parental lines are very different, early generation selection must be conducted toward optimal DH and PH so that in subsequent generations, the difference is minimized. By keeping progenies within the same optimal phenology, environmental selection will naturally follow toward drought and heat avoidance and resistance strategies (and drought escape will be ruled out). In this case, breeders will not be misleading selection toward escaping stress exclusively and will open and explore a new window of genetic variation. Similarly, any marker discovery efforts toward drought resistance and avoidance must use phenology controlled populations for adequate gene discovery.

Overall, to avoid the risk of limiting wheat yield progress in specific regions while exploring more than just drought escape in many parts of the world, the following strategy is suggested: (i) first define optimal heading and height ranges for the different target regions; (ii) select new progenies based on optimal heading and height defined in (i); (iii) compose yield trials compartmentalized in groups of wheat lines having optimal heading and height for the target regions defined in (i); and (iv) within each optimal heading and height group, identify the highest yielding lines which correspond to most resistant and avoidant lines.

Finally, this research revealed that the use of groups of genotypes (e.g., five genotypes) did not increase wheat resilience through compensation and adaptation mechanisms of different traits associated with different genotypes. In all tested sets of field trials, it was concluded that monoculture of the best selected genotype always demonstrated higher production.

## Author Contributions

ML: conception and design of the research, data collection with analysis and interpretation, critical revision of the article, and overall responsibility. CR, FA, MS-G, FO, MJ-K, and DP: critical revision of the article. MK, EO, and MR: conducted field trials, collected data, and critical revision of the article.

## Conflict of Interest Statement

The authors declare that the research was conducted in the absence of any commercial or financial relationships that could be construed as a potential conflict of interest.

## Supplementary Material

FIGURE S1 Heading and maturity date of a collection of 250 winter wheat varieties and breeding lines grown in eight trials conducted between 2012 and 2016 in Turkey (Diyarbakir and Konya) and Iran (Maragheh) (location and year indicated in the lower right corner of each diagram) during the wheat cycle (*xx* axes date). Daily maximum temperature (dark solid line), daily average temperature (dashed dark line), and daily minimum temperature (gray solid line) are all indicated in the primary *yy* axes and daily rain (light gray bars) are indicated in the secondary *yy* axes. Sowing time is indicated by the symbol “S,” days to heading of the entire population “H,” and days to maturity “M.”

TABLE S1 Pearson correlation coefficients and probability between grain yield (GY) and average temperature 20 days before and after heading (TA20AH and TA20BH), maximum temperature 20 days before and after heading (TM20AH and TM20BH), and minimum temperature 20 days before and after heading (Tm20BH and Tm20AH). Eight trials were conducted as indicated in **Table 1**. ^∗∗∗^, significant at *p* < 0.0005; ^∗∗^, significant at *p* < 0.005; ^∗^, significant at *p* < 0.05, NS, non-significant. Significant correlations are shown in bold values.

TABLE S2 Range of variation of optima (where grain yield is maximized) days to heading (DH OPTIM) and plant height (PH OPTIM) determined in each location at Diyarbakir for two growth seasons (DIY), Konya for three growth seasons (KON), Maragheh for three growth seasons (MAR), and across all (ALL) environments (from **Figures 3, 4**).

TABLE S3 Selection of wheat breeding lines showing optima combination of days to heading and plant height in Diyarbakir (DIY SEL) for 2 years (“U1275-1-4-2-2/KS85W663-7-4-2//JAGGER” accession number, ACCN# 30961), in Konya (KON SEL) for 3 years (“MADSEN/TAM-202//TX89V4138,” ACCN# 40662), and in Maragheh (MAR SEL) for 3 years (“WO405D/HGF112//W7469C/HCF012,” ACCN# 70546) and across all environments (ALL SEL) and years (“TAM 105/3/NE70654/BBY//BOW“S”/4/Century^∗^3/TA2450”ACC# 70554). Functional genes present (1) and absent (0) are shown. DHAVG, PHAVG, GYAVG: days to heading (DHAVG), plant height (PHAVG), and grain yield (GYAVG) average across all environments and years.

TABLE S4 Grain yield (GY) of genotypes selected based on optima days to heading (DH) and plant height (PH) in each location (Diyarbakir-DIY SEL for two seasons, Konya-KON SEL with for three seasons, and Maragheh-MAR SEL for three seasons) and across all locations and years (ALL). As a reference, the highest yielding genotype in each location is shown (HGY_ENV). Changes in GY between the two types of selection (selected in each location versus selected across all locations and years) are also shown in absolute values and in percentage (CHANGE_GY and % GY CHANGE, respectively).
