# Supplementary material for: Optimizing Winter Wheat Resilience to Climate Change in Rain Fed Crop Systems of Turkey and Iran
Source: Front Plant Sci. 2018 May 1;9:563. doi: 10.3389/fpls.2018.00563 (PMC5938555; doi:10.3389/fpls.2018.00563)
Supplement: FIGURE S1 — Heading and maturity date of a collection of 250 winter wheat varieties and breeding lines grown in eight trials conducted between 2012 and 2016 in Turkey (Diyarbakir and Konya) and Iran (Maragheh) (location and year indicated in the lower right corner of each diagram) during the wheat cycle (xx axes date). Daily maximum temperature (dark solid line), daily average temperature (dashed dark line), and daily minimum temperature (gray solid line) are all indicated in the primary yy axes and daily rain (light gray bars) are indicated in the secondary yy axes. Sowing time is indicated by the symbol “S,” days to heading of the entire population “H,” and days to maturity “M.” [file Data_Sheet_1.docx]

Supplementary Fig. 1
