# Supplementary material for: Optimizing Winter Wheat Resilience to Climate Change in Rain Fed Crop Systems of Turkey and Iran
Source: Front Plant Sci. 2018 May 1;9:563. doi: 10.3389/fpls.2018.00563 (PMC5938555; doi:10.3389/fpls.2018.00563)
Supplement: TABLE S1 — Pearson correlation coefficients and probability between grain yield (GY) and average temperature 20 days before and after heading (TA20AH and TA20BH), maximum temperature 20 days before and after heading (TM20AH and TM20BH), and minimum temperature 20 days before and after heading (Tm20BH and Tm20AH). Eight trials were conducted as indicated in Table 1. ∗∗∗, significant at p < 0.0005; ∗∗, significant at p < 0.005; ∗, significant at p < 0.05, NS, non-significant. Significant correlations are shown in bold values. [file Table_1.docx]

Supplementary Table 1- Pearson correlation coefficients and probability between grain yield (GY) and average temperature 20 days before and after heading (TA20AH and TA20BH), maximum temperature 20 days before and after heading (TM20AH and TM20BH), minimum temperature 20 days before and after heading (Tm20BH and Tm20AH). Eight trials were conducted as indicated in Table 1. *** significant at p<0.0005; ** significant at p<0.005; * significant at p<0.05, ns, non significant. Significant correlations are shown in bold values

| ENV | TA20BH | TA20AH | TM20BH | TM20AH | Tm20BH | Tm20AH | RAINVEG | RAINGF |
| --- | --- | --- | --- | --- | --- | --- | --- | --- |
| 1 | -0.10  NS | **0.15**  ****** | **0.15**  ****** | 0.09  NS | **0.26**  ******* | **-0.15**  ****** | **-0.19**  ****** | **0.24**  ******* |
| 2 | -0.11  NS | -0.12  NS | -0.12  NS | -0.12  NS | **-0.13**  ***** | **-0.13**  ***** | **-0.32**  ******* | **0.37**  ******* |
| 3 | **0.16**  ***** | **0.16**  ***** | **0.16**  ***** | **0.14**  ***** | **0.18**  ***** | **0.15**  ***** | 0.11  NS | 0.06  NS |
| 4 | 0.04  NS | 0.07  NS | 0.03  NS | 0.07  NS | 0.08  NS | 0.04  NS | -0.11  NS | 0.10  NS |
| 5 | **-0.36**  ******* | **-0.37**  ******* | **-.36**  ******* | **-.37**  ******* | **-0.19**  ****** | **-0.35**  ******* | **-0.23**  ****** | **0.17**  ****** |
| 6 | **-0.34**  ******* | **-0.33**  ******* | **-0.35**  ******* | **-0.31**  ******* | **-0.38**  ******* | **-0.31**  ******* | **-0.38**  ******* | **0.33**  ******* |
| 7 | 0.02  NS | 0.07  NS | -0.10  NS | 0.08  NS | 0.09  NS | 0.05  NS | -0.08  NS | -0.05  NS |
| 8 | **-0.23**  ****** | **-0.22**  ****** | **-0.22**  ****** | **-0.20**  ***** | **-0.24**  ****** | **-0.23**  ****** | **-0.15**  ***** | **0.15**  ***** |
