# Supplementary material for: Optimizing Winter Wheat Resilience to Climate Change in Rain Fed Crop Systems of Turkey and Iran
Source: Front Plant Sci. 2018 May 1;9:563. doi: 10.3389/fpls.2018.00563 (PMC5938555; doi:10.3389/fpls.2018.00563)
Supplement: TABLE S2 — Range of variation of optima (where grain yield is maximized) days to heading (DH OPTIM) and plant height (PH OPTIM) determined in each location at Diyarbakir for two growth seasons (DIY), Konya for three growth seasons (KON), Maragheh for three growth seasons (MAR), and across all (ALL) environments (from Figures 3, 4). [file Table_2.docx]

Supplementary Table 2-Range of variation of optima (where grain yield is maximized) days to heading (DH OPTIM) and plant height (PH OPTIM) determined in each location at Diyarbakir for two growth seasons (DIY), Konya for three growth seasons (KON), Maragheh for three growth seasons (MAR) and across all (ALL) environments (from Fig. 3 and 4).

|  | DH OPTIM (Days) | PH OPTIM (cm) |
| --- | --- | --- |
| DIY | 130.2±-4.8 | 94.4±15.4 |
| KON | 203.1±2.7 | 67.6±9.4 |
| MAR | 229.4±1.9 | 72.3±6.0 |
| All | 194.9±1.8 | 76.0±5.9 |
