# Supplementary material for: Optimizing Winter Wheat Resilience to Climate Change in Rain Fed Crop Systems of Turkey and Iran
Source: Front Plant Sci. 2018 May 1;9:563. doi: 10.3389/fpls.2018.00563 (PMC5938555; doi:10.3389/fpls.2018.00563)
Supplement: TABLE S3 — Selection of wheat breeding lines showing optima combination of days to heading and plant height in Diyarbakir (DIY SEL) for 2 years (“U1275-1-4-2-2/KS85W663-7-4-2//JAGGER” accession number, ACCN# 30961), in Konya (KON SEL) for 3 years (“MADSEN/TAM-202//TX89V4138,” ACCN# 40662), and in Maragheh (MAR SEL) for 3 years (“WO405D/HGF112//W7469C/HCF012,” ACCN# 70546) and across all environments (ALL SEL) and years (“TAM 105/3/NE70654/BBY//BOW“S”/4/Century∗3/TA2450”ACC# 70554). Functional genes present (1) and absent (0) are shown. DHAVG, PHAVG, GYAVG: days to heading (DHAVG), plant height (PHAVG), and grain yield (GYAVG) average across all environments and years. [file Table_3.docx]

Supplementary Table 3-Selection of wheat breeding lines showing optima combination of days to heading and plant height in Diyarbakir (DIY SEL) for two years (“U1275-1-4-2-2/KS85W663-7-4-2//JAGGER” accession number, ACCN# 30961), in Konya (KON SEL) for three years (“MADSEN/TAM-202//TX89V4138”, ACCN# 40662) and in Maragheh (MAR SEL) for three years (“WO405D/HGF112//W7469C/HCF012”, ACCN# 70546) and across all environments (ALL SEL) and years (“TAM 105/3/NE70654/BBY//BOW"S"/4/Century*3/TA2450”ACC# 70554). Functional genes present (1) and absent (0) are shown.

|  |  | DIY SEL | KON SEL | MAR SEL | ALL SEL |
| --- | --- | --- | --- | --- | --- |
|  | ACCN# | 30961 | 40662 | 70546 | 70554 |
| gene | allele |  |  |  |  |
| Rht-B1 | Rht-B1b (Rht1) | 1 | 1 | 1 | 1 |
| Rht-D1 | Rht-D1b (Rht2) | 0 | 0 | 0 | 0 |
| Vrn-A1(promoter) | Vrn-A1a (A) | 0 | 0 | 0 | 0 |
|  | vrn-A1(winter, G) | 1 | 1 | 1 | 1 |
| Vrn-A1 (SNP in exon7 | C (earlier flowering) | 0 | 1 | 1 | 1 |
| Vrn-B1 | Vrn-B1a | 0 | 0 | 0 | 0 |
|  | Vrn-B1b | 0 | 0 | 0 | 0 |
|  | Vrn-B1c | 0 | 0 | 0 | 0 |
|  | vrn1 (winter) | 1 | 1 | 1 | 1 |
| Vrn-D1 | Vrn-D1a | 0 | 0 | 0 | 0 |
| Ppd-B1 | Ppd-B1a (inse. 3x copy) | 1 | 1 | 0 | 0 |
|  | Ppd-B1b (sensitive | 0 | 0 | 1 | 1 |
| Ppd-D1 | Ppd-D1a (T, insensitive) | 0 | 0 | 1 | 1 |
|  | Ppd-D1 (c, wt) | 1 | 1 | 0 | 0 |
|  | DHAVG | 193.0 | 194.569 | 195.7 | 193.9 |
|  | PHAVG | 74.1 | 69.769 | 71.8 | 75.8 |
|  | GYAVG | 304.6 | 333.427 | 309.4 | 334.8 |

DHAVG, PHAVG, GYAVG: days to heading (DHAVG), plant height (PHAVG) and grain yield (GYAVG) average across all environments and years.
