# Supplementary material for: Optimizing Winter Wheat Resilience to Climate Change in Rain Fed Crop Systems of Turkey and Iran
Source: Front Plant Sci. 2018 May 1;9:563. doi: 10.3389/fpls.2018.00563 (PMC5938555; doi:10.3389/fpls.2018.00563)
Supplement: TABLE S4 — Grain yield (GY) of genotypes selected based on optima days to heading (DH) and plant height (PH) in each location (Diyarbakir-DIY SEL for two seasons, Konya-KON SEL with for three seasons, and Maragheh-MAR SEL for three seasons) and across all locations and years (ALL). As a reference, the highest yielding genotype in each location is shown (HGY_ENV). Changes in GY between the two types of selection (selected in each location versus selected across all locations and years) are also shown in absolute values and in percentage (CHANGE_GY and % GY CHANGE, respectively). [file Table_4.docx]

Supplementary Table 4-Grain yield of genotypes selected based on optima days to heading (DH) and plant height (PH) in each location (Diyarbakir- DIY SEL for two seasons, Konya-KON SEL with for three seasons and Maragheh- MAR SEL for three seasons) and across all locations and years (ALL). As a reference, the highest yielding genotype in each location is shown (HGY_ENV). Changes in grain yield between the two types of selection (selected in each location versus selected across all locations and years) are also shown in absolute values and in percentage (CHANGE_GY and % GY CHANGE, respectively).

|  |  | YEAR 1 |  |  | YEAR 2 |  | YEAR 3 |  |  |
| --- | --- | --- | --- | --- | --- | --- | --- | --- | --- |
|  | ACCN# | DIY | KON | MAR | KON | MAR | DIY | KON | MAR |
|  |  |  |  |  | gm^-2^ |  |  |  |  |
| DIY SEL | 30961 | 494.3 | 495.5 | 87.5 | 113.1 | 242.0 | 432.0 | 324.1 | 231.8 |
| KON SEL | 40662 | 228.2 | 554.1 | 126.3 | 247.7 | 292.3 | 430.6 | 455.0 | 333.1 |
| MAR SEL | 70546 | 398.8 | 397.6 | 160.8 | 142.6 | 292.3 | 348.0 | 337.8 | 419.4 |
| ALL | 70554 | 466.5 | 486.0 | 126.7 | 175.2 | 300.6 | 376.9 | 410.3 | 305.0 |
| HGY_ENV (gm^-2^) | | 580.5 | 638.9 | 171.0 | 250.2 | 369.8 | 475.5 | 524.4 | 419.4 |
| CHANGE_GY(gm^-2^) | | 27.8 | 68.1 | 34.1 | 72.5 | -8.2 | 55.1 | 44.8 | 114.4 |
| % CHANGE_GY | | 6.0 | 14.0 | 27.0 | 41.4 | -2.7 | 14.6 | 10.9 | 37.5 |
